# Feeding in predator naïve crayfish is influenced by cues from a fish predator

**DOI:** 10.1038/s41598-023-39406-w

**Published:** 2023-07-28

**Authors:** Martin Musil, Marek Let, Sara Roje, Bořek Drozd, Antonín Kouba

**Affiliations:** Faculty of Fisheries and Protection of Waters, South Bohemian Research Center of Aquaculture and Biodiversity of Hydrocenoses, University of South Bohemia in České Budějovice, Zátiší 728/II, 389 25 Vodňany, Czech Republic

**Keywords:** Invasive species, Animal behaviour, Biodiversity

## Abstract

In this study, we experimentally evaluated how the feeding behaviour of marbled crayfish *Procambarus virginalis* is influenced by cues from conspecifics and the round goby *Neogobius melanostomus*, a fish predator, in tanks that permitted chemical communication but not visual recognition. We used four experimental groups with different combinations in two sub-tanks. The first sub-tank always contained a crayfish and prey (40 individuals of the water louse *Asellus aquaticus*). The other sub-tanks were set up as follows: (i) empty, serving as a control (C); (ii) with a conspecific crayfish (Cr); (iii) with a round goby (G) to simulate predator-only odour; and (iv) a round goby and three small conspecific crayfish (G + Cr) to simulate the presence of a predator and/or the alarm odour. Two sub-treatments were defined for the fourth group, categorised as ‘injured’ or ‘not injured’ depending on whether prey crayfish were visibly injured or not, respectively. We observed a significant decline in the consumption of water lice in the G and G + Cr treatments compared to the C and Cr treatments (up to 47% on average). There were no significant differences in consumption between the G and G + Cr treatments, or C and Cr treatments. No significant differences in food consumption parameters were detected between sub-treatments with ‘injured’ and ‘not injured’ conspecific crayfish. Knowledge of modifications in the feeding behaviour of marbled crayfish in the presence of round goby (and fish predators in general) is essential for ecologists attempting to understand the changes and impacts occurring in invaded freshwater ecosystems.

## Introduction

Fish and crayfish play prominent roles in freshwater ecosystems^1–3^ where they co-exist in an ecological balance determined by predation, competition and habitat use^4^. Many studies have shown that established populations of non-native crayfish species change native crayfish behaviour and reduce their populations^5^. Established populations of non-native crayfish species, such as the North American spiny-cheek crayfish *Faxonius limosus*, signal crayfish *Pacifastacus leniusculus* and red swamp crayfish *Procambarus clarkii* are found all over Europe^6^; all these species are listed as invasive species of Union concern^7,8^. Other non-native crayfish species are increasing in number and range and negatively impacting native biota and invaded ecosystems^9–11^. Of all the freshly established non-native crayfish species in Europe, the parthenogenetic marbled crayfish *Procambarus virginalis* has spread most rapidly in European water bodies^11^ and is now also classified as invasive species of Union concern^7,8^.

Freshwater prey species typically rely on chemical signals to evaluate the risk of predation^12^. Chemicals indicating possible dangers are produced by two types of kairomones: alarm kairomones are given off by injured prey^13^, while predators emit predator kairomones^14^. As odours, alarm kairomones provide less reliable information about the predation risk than predator kairomones^15^. Prey can theoretically recognise predator species by their unique odour, which allows them to respond with appropriate defensive behaviour^16^. Predators release chemicals such as digestive enzymes or digestion by-products when they chew or digest prey^17,18^ and in their excreta^19^. Excreta are reliable odour indicators of the potential danger if they contain residues of prey items belonging to the same or closely related species^20^. Preys that perceive these cues are more vulnerable to generalist predators that frequently change their diet^21^. In some cases, odours emanate from a predator’s skin, which enables prey to detect the proximity of a predator in real-time, compared to other types of predator odours, such as those emitted from excreta or prey remains^22^.

Another invasive species is the round goby *Neogobius melanostomus*. This fish, native to the Ponto-Caspian region^23^, is spreading through European rivers^24–26^ and into coastal waters, as well as in North American freshwater ecosystems^27,28^. Expansion via new canal systems in Europe^29^ due to the use of ballast water^30^ or human translocations^31^ has been reported. The round goby is primarily an opportunistic benthic feeder with a broad diet spectrum of invertebrates, including crayfish^28^. Its ecological impact seriously affects invaded water bodies and their native biota^32^, including their native ecological counterparts such as the protected European bullhead *Cottus gobio*^33,34^. Populations of round goby in the Czech Republic are well established in the Morava^35^ and the Elbe^36^ river basins, where they live in syntopy with spiny-cheek crayfish (personal field observations). Due to releases of originally pet-traded marbled crayfish into the wild^37^, several populations of this crayfish already occur in the region^38^ and it is to be expected that they will come into direct contact in the future. In Hungary, this has occurred in certain places^9,39^ and so it follows that the syntopic occurrence of non-native crayfish with non-native round goby will increasingly take place throughout Europe, with as yet unknown mutual environmental interactions.

Fish predators modify the behaviour of their crayfish prey^40^, often by affecting the amount of time they spend sheltering and foraging^41^. Time spent sheltering and foraging by prey represents useful information for ecologists as fluctuations in these behavioural parameters will greatly affect interactions between prey and other species in the community^42^. For instance, the European perch *Perca fluviatilis* and eel *Anguilla anguilla* as predators decrease native noble crayfish *Astacus astacus* and invasive signal crayfish activity and lengthen the time they spend in their refuges^43,44^. Other studies have shown that red swamp crayfish respond to the alarm odour of conspecifics^45^, as do other crayfish species, including the white-clawed crayfish *Austropotamobius pallipes*, rusty crayfish *Faxonius rusticus* and virile crayfish *Faxonius virilis*^46^.

The threat-sensitive predator avoidance hypothesis advanced by Helfman^47^ states that a prey organism considers various factors when classifying the danger from a potential predator and takes appropriate action in terms of the perceived risk. Detailed behavioural information about how crayfish as prey use chemical cues emitted by predators to evaluate the danger in the surrounding environment—and, above all, how food consumption is affected by the presence of a fish predator—is still lacking. Crayfish are useful model organisms for such research because they are highly sensitive to predator and alarm odours^48–50^. Our study was based on an experimental evaluation of the food consumption rate in the invasive marbled crayfish in the presence of the cues from conspecifics and/or odours emitted by a predator, the round goby. Understanding how the marbled crayfish alters their feeding behaviour in response to the presence of a round goby (and fish predators in general) will help ecologists and conservation managers improve their knowledge of mutual interferences in these freshwater invaders and assess the changes and impacts occurring in invaded freshwater ecosystems.

## Materials and methods

### Experimental animals acquisition and maintenance

The round gobies were captured in the river Elbe (50° 50′ 37.2″ N, 14° 13′ 01.5″ E) using a backpack electrofishing unit (FEG 1500, EFKO, Leutkirch, Germany). The marbled crayfish, an all-female population, were obtained from our laboratory, and were therefore na﻿ïve individuals without any previous experience with fish predators. The water lice *Asselus aquaticus* were used as native benthic prey impacted by both mentioned fish and crayfish species at localities where they co-occur. They were collected in the Příbramský brook (49° 42′ 38.2″ N, 14° 00′ 30.3″ E) with fine nets and sorted manually. All animals were kept indoors in separate units from September 2020 until use in a small recirculating water system for acclimatisation to laboratory conditions at the Experimental Fish Culture Facility, Research Institute of Fish Culture and Hydrobiology in Vodňany, University of South Bohemia in České Budějovice (49° 09′ 14.8″ N, 14° 10′ 08.7″ E). All animals were kept under identical experimental light regime of 12L:12D, at 20 °C and with oxygen levels no lower than 8 mg l^−1^. Both fish and crayfish were fed daily with defrosted chironomid larvae. Crayfish were additionally provided with carrots and water lice from commercial fish feed (Sera Granugreen Nature, Germany).

All experimental animals were weighed using a digital precision scale (Kern 572–35, Kern and Sohn, Germany). Crayfish carapace lengths (CL, distance from the tip of the rostrum to the posterior median edge of the cephalothorax) were measured with a Vernier calliper; total fish lengths (TL, distance from the tip of the snout to the tip of the caudal fin) were measured with a ruler to the nearest 1 mm.

The TL (mean ± SD) and weight (W; mean ± SD) of all gobies used during the experiment were 112.8 ± 2.8 mm and 18.2 ± 0.9 g, respectively. All 120 crayfish individuals used for the experiment were size-matched, with an average CL (mean ± SD) = 20.7 ± 0.1 mm and weight W = 2.2 ± 0.1 g. Only individuals with intact appendages and fully hardened exoskeleton (intermoult phase) were used. Smaller crayfish used as prey for gobies had CL = 16.6 ± 0.1 mm and W = 1.2 ± 0.02 g. As prey, we used similar-sized water lice, with an average W of 40 individuals 0.586 ± 0.032 g. All animals were used for behavioural experiments only once.

No specific permissions were required for the locations and activities involved in this study. All manipulations with organisms were approved by the Institutional Animal Care and Use Committee (IACUC) of the University of South Bohemia, Faculty of Fisheries and Protection of Waters, Research Institute of Fish Culture and Hydrobiology, Vodňany, based on the EU harmonized animal welfare act of Czech Republic. The principles of laboratory animal care and the national laws 246/1992 and regulations on animal welfare were followed (Ref. number 22761/2009-17210). This study followed Arrive guidelines (https://arriveguidelines.org).

### Experimental arena and design

**Figure 1:**
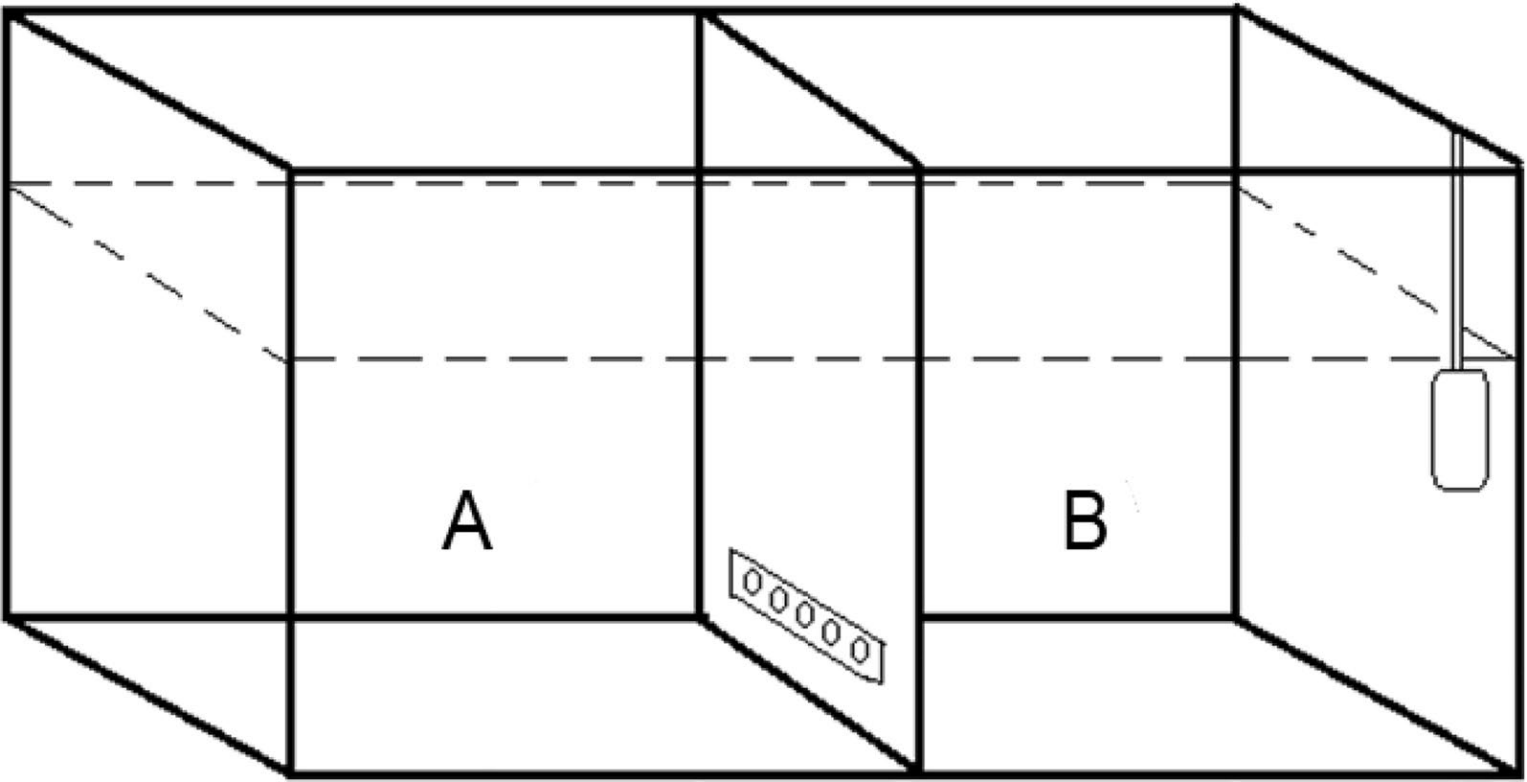
Experimental tank. Sub-tank A corresponds to the sector where the tested crayfish individual with prey was placed, while sub-tank B corresponds to the part in which the different experimental groups were kept (C, Cr, G or G + Cr). Aeration by aquarium pumps in the corner maintained suitable and stable dissolved oxygen concentrations and ensured that water flowed through the holes to connect the two sub-tanks.

The experiment was performed on 2–11 December 2020. To assess the influence of the predator and/or conspecific alarm odour on the food consumption rate of the marbled crayfish, ten separate two-hour trials (30 replicates for each treatment) during the day-time regime (500 lx m^−2^) were performed between 09.00 and 11.00. The acclimatisation of both the fish and crayfish in the experimental tanks lasted for 17 h from 16.00 on the day before the experiment until the start of the experiment. For crayfish, these 17 h also served as exposure time to treatment cues. No additional feed was added during acclimatization—exposure. The trial started after prey stocking. Experimental aquaria (40 × 20x25 cm) filled with 12 L of aerated tap water (20 °C) were used as experimental tanks. No connection via any water flow or visual recognition between the tanks was possible. Each tank was divided into two sub-tanks by an opaque plastic barrier positioned vertically in the middle to prevent visual recognition. Perforation (five 10-mm holes covered by a thin net located 20 mm from the bottom of the tank) allowed chemical communication between experimental animals to occur (see Fig. 1). No bottom substrate was provided. After every trial, the water was changed, and tanks were cleaned with hot water to remove any odours that might affect the following trial.

Sub-tank A was uniformly stocked with one crayfish and live prey (the initial number was 40), while sub-tank B was stocked according to one of the four different treatments: (i) empty, serving as control (C); (ii) with a conspecific crayfish individual (Cr); (iii) with a round goby (G) to simulate predator-only odour; or (iv) with a round goby and three small conspecific crayfish as prey (G + Cr) to simulate the presence of a predator and/or alarm odour of scared or injured prey. Two sub-treatments were further defined for the G + Cr group after each trial: ‘injured’ or ‘not injured’. The former classification corresponds to a sub-treatment with at least one injured or eaten small prey crayfish (with visible attack marks), while the latter refers to a sub-treatment in which all small prey crayfish remained alive with no visible signs of attack.

During each trial, three replicates of each treatment (C, Cr, G and G + Cr) and one experimental tank (stocked with 40 water lice to monitor the potential natural mortality of prey) were performed. The mortality of water lice was tested only for the control treatment type (predators and crayfish absent in an experimental tank). We monitored and evaluated the feeding rate of the tested crayfish using a modified version of the methodology employed by Veselý et al.^51^ as the number of *survived*, *eaten*, *killed* (specimens with visible attack marks) or *dead* (specimens with no visible attack marks) water lice. The number of *survived* water lice can indicate whether the crayfish are actively preying on the lice or avoiding them altogether. The number of *eaten* water lice indicates the overall feeding rate of the crayfish. This category provides information on how many water lice are consumed by crayfish and can help assess their feeding behaviour and efficiency. The number of *killed* water lice (specimens with visible attack marks) provides information on the predatory behaviour of the crayfish, indicating that the crayfish are actively hunting and attacking the lice, but not consuming them. Finally, the number of *dead* water lice (specimens with no visible attack marks) can provide information on whether the crayfish are directly responsible for the death of the lice, or whether they died from other causes such as stress. By assessing all four categories of water lice, the tested crayfish's feeding behaviour and predatory efficiency can be better understood, providing more detailed insights into their behaviour and ecology.

### Data analysis

The statistical analyses were conducted with R studio software (R Development Core Team, v. 3.6.1., 2019). For all statistical tests, *α* < 0.05 was applied. All results are presented as a mean or fitted probability ± standard error. To test for differences in the CL and W of crayfish between treatments (C, Cr, G and G + Cr), we used a Generalised Linear Model (GLM) with a gamma distribution for residual variation with logarithm as the link function. The statistical significance of differences between treatments (C, Cr, G and G + Cr) for ordinal response variables (number of *survived*, *eaten*, *killed* and *dead* water lice as a proportion of the initial number) was tested using a Cumulative Link Mixed Models (CLMM) with the logistic link function^52^. The identities of aquaria were used as a factor with a random effect on the intercept, and the nominal number of water lice (40) was used as weights. We also tested the relationship between the CL and the ordinal response variables, as well as the interaction between treatment and CL using the same method. We tried to improve the model with random effects of CL on slopes or intercepts; however, these models failed to be significantly better than the model with treatments and CL used as fixed-effect predictors (*p* > 0.05 according to a Likelihood Ratio Test). We conducted a CLMM^52^ to analyse the G + Cr treatment, testing for differences between two sub-treatments, ‘injured’ and ‘not injured’. We also tested the viability of water lice with one sample lower-tailed *t*-test; proportions of viability were transformed using the arcsine square root transformation.

## Results

The size of the tested crayfish individuals was uniform (CL 20.7 ± 0.1, 17–25 mm) and did not differ between tested groups (CL: *F*_3,110_ = 0.9, *p* = 0.44; W: *F*_3,110_ = 0.83, *p* = 0.83). The mortality of the water lice in the control tanks monitoring prey viability was 0.6 ± 0.4 individuals (*t*_9_ = −1.8; *p* = 0.052). Therefore, natural mortality was regarded as negligible regarding its possible effects on the mortality rate of lice with crayfish present as predators.

### Comparison of food consumption between treatments

**Figure 2:**
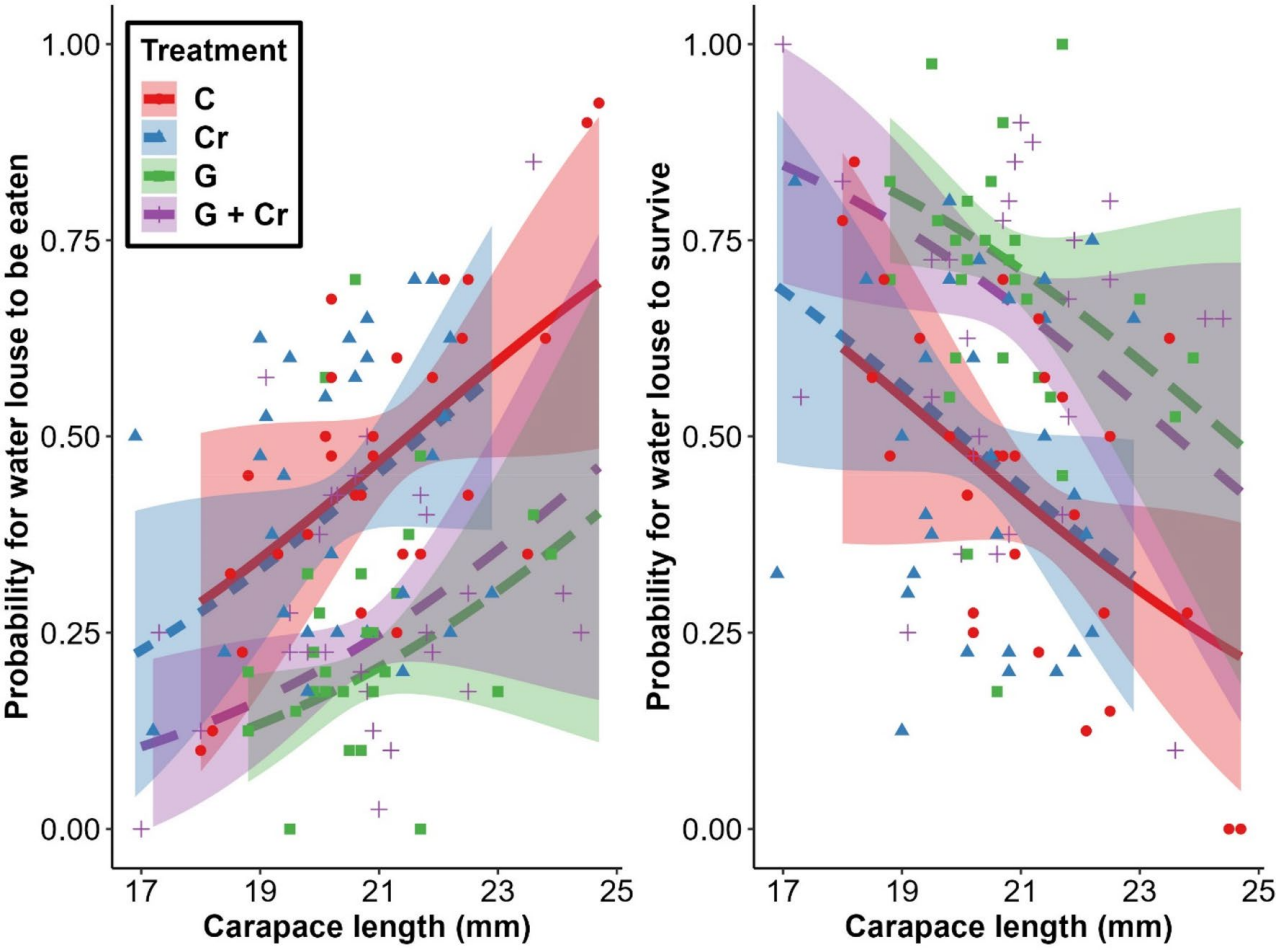
Significant effects of the treatment factor on (**a**) the probability of *eaten* water lice (graph on the left) and (**b**) the probability of *survived* water lice (graph on the right) in terms of the continuous variable ‘carapace length’ (CL), which was a significant predictor of both (**a**) and (**b**). The initial density of the water lice was 40 individuals. Cumulative Link Mixed Model with a logistic link function was used. Shaded areas represent the standard error for the CL gradient. Treatments: C = Control, Cr = Crayfish, G = Goby, and G + Cr = Goby with crayfish.

**Figure 3:**
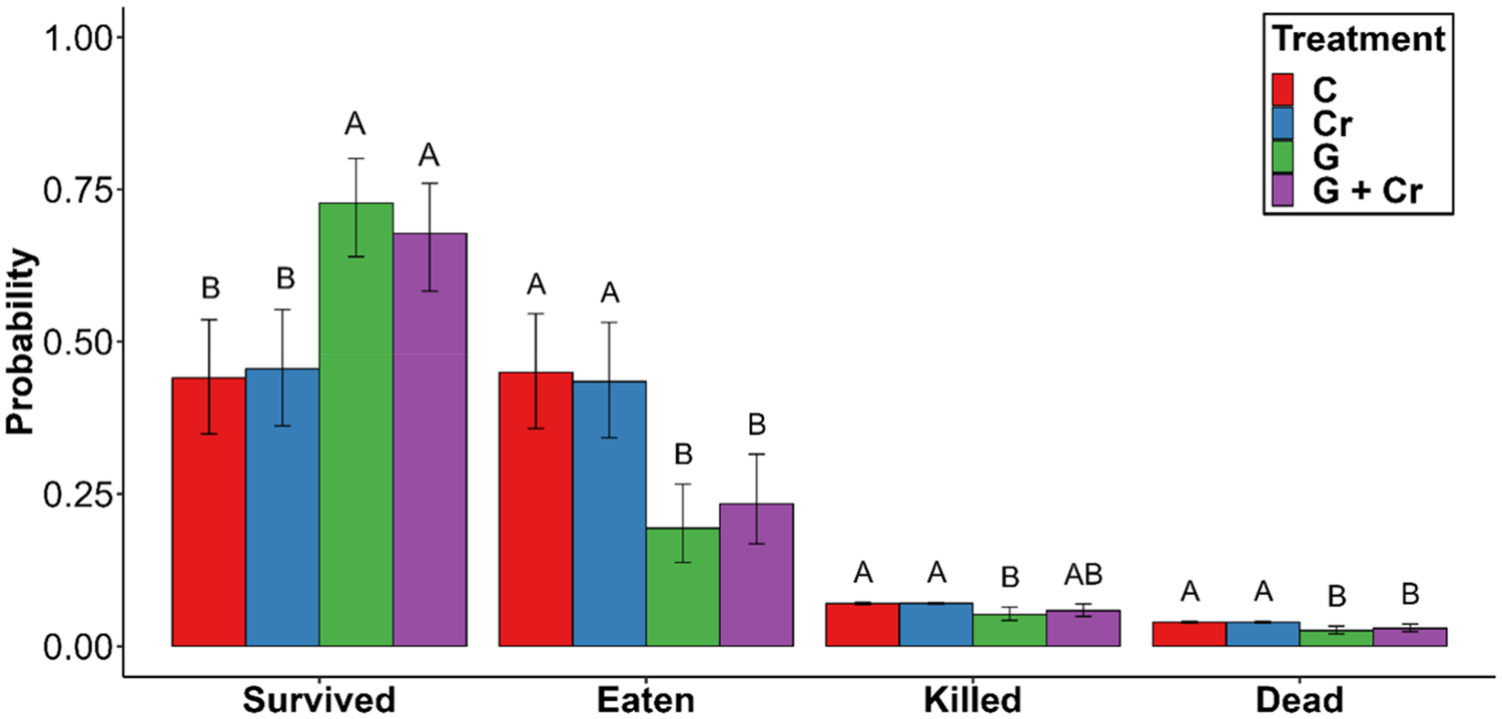
Probabilities of the number of (i) s*urvived*, (ii) *eaten*, (iii) *killed* and (iv) *dead* water lice for the different treatments for mean value of ‘carapace length’. The initial density of water lice was 40 individuals. Different letters (A and B) above each column denote significant differences between treatments for the given response parameters (*p* < 0.05) based on the 95% confidence intervals represented by ‘whiskers’. Treatments: C = Control, Cr = Crayfish, G = Goby, and G + Cr = Goby with crayfish.

The general test of significance between all treatments revealed different (*p* < 0.05) categories in lice (*χ*^2^_3_ = 25.42, *p* < 0.001). CL was also significantly linearly dependent (*χ*^2^_1_ = 14.74, *p* < 0.001). Interaction between treatment and CL was not detected (*χ*^2^_3_ = 4.33, *p* = 0.23; Fig. 2). Comparisons of water lice *eaten* between treatments indicate that crayfish consumed lice significantly more when the goby was absent (Fig. 3).

Of the initial total of 40 water lice, the highest probability of water louse to be *eaten* was 44.96 ± 4.86% in group C. A comparable but lower probability of water louse to be *eaten*—43.43 ± 4.88%—occurred in group Cr. Based on the 95% confidence intervals, these two groups did not statistically differ from each other (Fig. 3). However, significantly lower probability of water louse to be *eaten* was observed in groups G and G + Cr, 19.38 ± 3.26% and 23.38 ± 3.74%, respectively. These two groups did not statistically differ from each other (Fig. 3).

### Comparison of ‘injured’ and ‘not injured’ sub-treatments

In total, 40% of juvenile crayfish (n = 12) from the treatment G + Cr were in the ‘injured’ sub-treatments and 60% (n = 18) in the ‘not injured’ sub-treatments. In the ‘injured’ sub-treatments, 12.6 ± 2.6 individuals of water lice (31.5%) were eaten, whereas, in the ‘not injured’ sub-treatments, we recorded 11 ± 1.5 individuals of water lice (27.5%) as *eaten*. However, there were no significant differences between these sub-treatments (*χ*^2^_1_ = 0.29, *p* = 0.59), while the CL was a significant predictor (*χ*^2^_1_ = 4.15, *p* = 0.04). Interaction between sub-treatment and CL was marginally significant (*χ*^2^_1_ = 3.52, *p* = 0.06).

## Discussion

European freshwater ecosystems have been invaded by numerous non-native crayfish species, some of which are now widely distributed throughout the continent^6^. Of these new arrivals, the marbled crayfish is particularly problematic and is currently found in several localities in the Czech Republic^6,38^. Moreover, another freshwater invader—the round goby, an opportunistic benthic fish predator that feeds on crayfish^28^—has also recently formed stable populations in the same regions of the country^35,36^. Therefore, their future co-occurrence can be expected to occur in the Czech Republic, as has already happened in neighbouring Hungary^9,39,53^.

Interactions between crayfish and fish, including competition for shelter, direct predation and behavioural alterations have previously been described^54^. Crayfish are a known prey item of fish^2,55,^ and studies show that the round goby successfully predates hard-bodied prey species such as marbled crayfish^56^, spiny-cheek crayfish^54^ and signal crayfish; especially the size of the goby’s mouth gape acts as a limiting factor for swallowing crayfish^57^. However, we lack knowledge of the behaviour of crayfish as prey when they detect the chemical cues emitted by fish predators, which help them evaluate the degree of danger in the environment. Foraging behaviour, including food consumption rates, is particularly poorly studied. However, there may be a relative paucity of research on indirect effects on other macroinvertebrates due to changes in macroinvertebrate consumption by crayfish in response to predatory threats. Therefore, further research in this specific field could help to shed more light on the complex interactions between different species in aquatic ecosystems and the potential cascading effects of changes in predator behaviour on other species.

Our study proved that crayfish food consumption was significantly reduced by the presence of the odours emitted by a fish predator, independently of the presence/absence of other crayfish individuals that could also act as prey. Thus, it follows that crayfish did not respond to the alarm odours produced by scared or injured conspecifics by reducing their food intake. However, a comparison with the findings of Gherardi et al.^58^, where the results were exactly the opposite, may not be entirely accurate. Our experiment did not test alarm odours in isolation from predator kairomones, so it is difficult to determine the specific effects of each type of odour on crayfish behaviour. Further research would be needed to investigate the specific effects of alarm odours and predator kairomones on crayfish behaviour, potentially by testing each type of odour in isolation or in different combinations. Moreover, the presence of conspecific crayfish did not significantly affect their food consumption rates, which were comparable with the control group in which no fish predators were present (Fig. 3). Plasticity in crayfish behaviour is well-known^59,60^. According to Acquistapace et al.^45^, crayfish originating from aquaculture ponds, i.e. an environment in which the predation risk is low, showed no fright responses after exposure to alarm odour. This assertion is also supported by Gherardi et al.^58^, who confirm that crayfish categorise by odour intensity the risk they face from fish predators, and that their ability to feel fear is not innate. Other studies on crayfish suggest that recognising predator odours may depend on learned rather than innate responses^61,62^. For instance, according to Hazlett et al.^46^, red swamp crayfish can recognise a predatory fish after a single exposure to a predator’s odour, and this cue is remembered without refreshing for up to three weeks. Yet, despite being predator-naïve, the marbled crayfish individuals we used in our study did respond to predator odours. This refutes theories that state that the ability to feel fear is not innate and that na﻿ïve crayfish should show no fright responses. Animals responding to predatory threats experience an increase in their stress hormone levels^63,64^, which represents a high metabolic cost, and stimulates greater food intake^65^. According to Wood et al.^50^, this phenomenon also occurs in crayfish as individuals exposed to predator odours feed more than non-exposed ones. This strong indirect effect of predatory fish odour on crayfish foraging behaviour—i.e., modifying their dietary preferences—could significantly negatively impact the faunal composition of lotic waters, including macrophyte communities^50^. While our study did not explicitly measure changes in dietary preferences, it is possible that a shift in foraging behaviour indirectly affects the types of organisms that crayfish consume, as this may depend on a variety of factors such as the availability of alternative food sources and the magnitude of the odour cues. However, it is worth noting that our study did find a significant reduction in overall foraging activity in the presence of predator odours, which suggests that this effect could potentially have significant ecological implications. Gherardi et al.^58^ assert that crayfish reduce their feeding rates more in response to a conspecific alarm odour than to a predator odour. However, in our study, in the treatment with both round goby and small prey crayfish, which was divided into two sub-treatments according to the categorisation of prey crayfish as ‘injured’ or ‘not injured’, no significant differences between the consumption parameters were detected (see part 3.2. Comparison of ‘injured’ and ‘not injured’ sub-treatments). This was most likely because the predator odour was more intense than the conspecific alarm odour derived from the scared and/or injured prey crayfish, whose concentrations or effects were insufficient to induce a change in crayfish foraging behaviour, as has previously been documented in the virile crayfish by Ramberg-Pihl and Yurewicz^66^. These authors also describe how crayfish in an alarm cue treatment moved around in an experimental tank significantly less than individuals in a predator cue treatment. This is supported by other studies in which crayfish reduced their activity after exposure to alarm cues^45,67^. Although emanate alarm cues can indicate the presence of danger, they may not provide as much information about the specific nature or level of the threat as compared to predator odours^68^. However, these findings could be interpreted in other ways as the responses in crayfish as prey to predator stimuli are a complex and individual-dependent issue whose outcome is hardly predictable. For example, previous experiences with predators, the presence of conspecifics, and the physical environment (such as the habitat's complexity) could affect how a crayfish responds to a predator stimulus. Additionally, genetic or other individual differences may contribute to variation in crayfish behaviour^69^. Our experimental setup simulated conditions of cryptic littoral microhabitats, i.e. natural ecosystems inhabited by crayfish, where only chemical stimuli and no visual recognition are often present. In these habitats, crayfish can also detect movements and vibrations through mechanoreceptors located on antennae and other appendages^70^. However, the extent to which this sensory capacity influences their ability to detect and discern prey or predators remains uncertain. As a result, this particular aspect was not included or investigated within the parameters of the present study. According to Bouwma and Hazlett^71^, visual recognition of predators does not affect responses to alarm odours, regardless of concentration. This suggests that the chemical recognition of potential predators by prey is a more important mechanism than visual recognition^62,72^, which is to be expected in cryptic nocturnal organisms like crayfish^73^.

Body size may be another important factor for predicting responses in crayfish as prey to predator odours^40^. Crayfish juveniles and smaller adults are theoretically more available and desirable prey items than larger individuals for gape-size limited fish predators^74^. According to Stein and Magnuson^75^, crayfish with CL < 23 mm significantly decreased their movement as a result of predator odours compared to larger specimens with CL > 26 mm. Keller and Moore^76^ also detected significantly stronger behavioural responses to predator odour in smaller crayfish. Nevertheless, Ramberg-Pihl and Yurewicz^66^ reported no significant effect of CL on crayfish locomotory responses to predator presence and chemical stimuli. We were unable to assess the effect of CL on food consumption rates because our tested crayfish were uniform in size (no significant difference between different treatments was found). Nevertheless, based on our data, we observed a general pattern of higher food consumption rates in crayfish with greater CL, independently of the treatment.

Given the natural complexities of predator–prey responses, several key requirements for future studies emerge, including the need to monitor the different responses of prey to predator presence by simultaneously assessing food consumption and video recordings of both predator and prey behaviour^69^. Understanding the response of the invasive marbled crayfish to the presence of the invasive round goby—and predatory fish in general—may help us understand, predict and possibly manage ongoing changes occurring in invaded freshwater ecosystems. The ethological effects of the presence of round goby, which include high feeding pressure on marbled crayfish^56^, competition for shelter, and other direct and indirect consequences of co-occurring invasive crayfish^11,54^, may result in behavioural alterations in crayfish and changes in invaded ecosystems. In addition to the aforementioned impacts, detailed accounts of additional impacts are reported in the literature. For instance, a comparative study examining the interactions between native European bullhead and non-native round goby describes the impacts in great detail^33^. Similarly, another study has explored the predation of round goby as a predator on marbled crayfish of varying size classes^57^. All the effects, including the lower food consumption in crayfish, shown in our study reflect the potential impact of invasive predatory fish on both native and non-native crayfish in freshwater ecosystems.

## Data Availability

All data generated or analysed during this study are included in this published article.
